# mTOR-Related Cell-Clearing Systems in Epileptic Seizures, an Update

**DOI:** 10.3390/ijms21051642

**Published:** 2020-02-28

**Authors:** Fiona Limanaqi, Francesca Biagioni, Carla Letizia Busceti, Cinzia Fabrizi, Alessandro Frati, Francesco Fornai

**Affiliations:** 1 Department of Translational Research and New Technologies in Medicine and Surgery, University of Pisa, Via Roma 55, 56126 Pisa, Italy; f.limanaqi@studenti.unipi.it; 2 I.R.C.C.S. Neuromed, Via Atinense 18, 86077 Pozzilli, Italy; francesca.biagioni@neuromed.it (F.B.); carla.busceti@neuromed.it (C.L.B.); alessandro.frati@uniroma1.it (A.F.); 3 Department of Anatomy, Histology, Forensic Medicine and Orthopedics, Sapienza University of Rome, Via A. Borelli 50, 00161 Rome, Italy; cinzia.fabrizi@uniroma1.it

**Keywords:** autophagy, proteasome, seizures, GABA, glutamate, dopamine, neurodevelopment

## Abstract

Recent evidence suggests that autophagy impairment is implicated in the epileptogenic mechanisms downstream of mTOR hyperactivation. This holds true for a variety of genetic and acquired epileptic syndromes besides malformations of cortical development which are classically known as mTORopathies. Autophagy suppression is sufficient to induce epilepsy in experimental models, while rescuing autophagy prevents epileptogenesis, improves behavioral alterations, and provides neuroprotection in seizure-induced neuronal damage. The implication of autophagy in epileptogenesis and maturation phenomena related to seizure activity is supported by evidence indicating that autophagy is involved in the molecular mechanisms which are implicated in epilepsy. In general, mTOR-dependent autophagy regulates the proliferation and migration of inter-/neuronal cortical progenitors, synapse development, vesicular release, synaptic plasticity, and importantly, synaptic clustering of GABA_A_ receptors and subsequent excitatory/inhibitory balance in the brain. Similar to autophagy, the ubiquitin–proteasome system is regulated downstream of mTOR, and it is implicated in epileptogenesis. Thus, mTOR-dependent cell-clearing systems are now taking center stage in the field of epilepsy. In the present review, we discuss such evidence in a variety of seizure-related disorders and models. This is expected to provide a deeper insight into the molecular mechanisms underlying seizure activity.

## 1. Introduction

Seizures are produced by abnormal, synchronous, high-frequency neuronal firing within the central nervous system [1]. Epilepsy is a chronic neurological disorder featuring recurrent, unprovoked seizures. The term epilepsy actually comprises a number of aetiologically diverse syndromes, which are associated with either inborn or acquired brain malformations, structural lesions, or other neurological disorders [2]. During epileptogenesis, that is, the process of converting a nonepileptic brain into one capable of generating spontaneous, recurrent seizures, a plethora of structural and cellular mechanisms occur, fostering persistently increased neuronal excitability and abnormal plasticity [3,4]. Neuronal hyperexcitability is mainly attributed to an imbalance between glutamate and gamma-aminobutyric acid (GABA) neurotransmitter systems, though alterations in catecholamine systems play a role in epileptogenesis as well [5].

Among the intracellular signaling pathways implicated in epileptogenesis, the mammalian target of rapamycin (mTOR) has received particular attention due to its multiple roles in brain development, neuronal excitability, and plasticity, as well as inflammation and immunity [6,7]. The key role of the mTOR pathway in epileptogenesis is best exemplified by tuberous sclerosis complex (TSC) and focal cortical dysplasia (FCD), the most common genetic causes of epilepsy [8]. As extensively documented in the literature, mTOR hyperactivation due to mutations in phosphatase and tensin homolog (*PTEN*) and *TSC1/2* genes leads to epileptogenesis in human samples and mouse models; conversely, inhibition of mTOR prevents the development of epilepsy and underlying neuronal alterations [9,10,11,12,13,14]. Accumulating evidence indicates that mTOR also participates in epileptogenesis associated with other forms of genetic or acquired epilepsy such as Lafora disease (LD), temporal lobe epilepsy (TLE), traumatic brain injury, and experimental epilepsy induced by chemoconvulsive compounds [11,12,13,14]. As recently addressed, mTOR pathway activation is also implicated in autoimmune diseases such as systemic lupus erythematosus (SLE), which represents a prominent cause of seizures [7]. Clinical cases of fulminant SLE have also been documented in TSC patients, supporting a key role of mTOR in seizure development associated with these disorders [15,16]. In detail, in SLE, mTOR acts as a critical driver of inflammatory lineage development of the immune system while fostering generation of antiphospholipid antibodies, which are mediators of seizure in adults and children alike [17,18].

An emerging mTOR-dependent mechanism contributing to epileptogenesis is bound to alterations of cell-clearing systems. This emerged mainly from evidence indicating that rapamycin, a powerful mTOR inhibitor and autophagy inducer, strongly modulates a variety of seizure models and epilepsies [12,13]. More recently, direct evidence has been provided indicating a solid correlation among mTOR-dependent autophagy, epileptogenesis and epilepsy-induced neuronal damage. In fact, similarly to autophagy suppression which occurs following mTOR hyperactivation, impaired autophagy due to the deficiency of ATG18 is linked to encephalopathic seizures [19,20], and ablation of ATG7 in mice models leads to spontaneous seizures [21]. These findings suggest that autophagy failure may be sufficient per se to induce epilepsy. In support of the role of autophagy disruption in the pathogenesis of epilepsy, autophagy alterations are detected in human samples and experimental models of epilepsy [21,22]. This casts the hypothesis that altered autophagy may contribute to the occurrence of epilepsy, and in turn, that epilepsy could impinge on the autophagy pathway, creating a vicious cycle which might further exacerbate epilepsy-induced neuronal damage. This would not be surprising since autophagy regulates a variety of cell functions that are implicated in neurodevelopmental and neurological disorders, including epilepsy. In fact, besides coping with harmful events such as oxidative damage and mitochondrial alterations, mTOR-dependent autophagy regulates the proliferation and migration of inter-/neuronal cortical progenitors, synapse development, axon guidance, dendritic spine architecture and pruning, vesicular release, and synaptic plasticity [23,24,25]. Once thought to play a merely housekeeping role by removing misfolded proteins or compromised organelles, neuronal autophagy is now regarded as a finely tuned surveillance system, which operates in neurons to guarantee synaptic integrity and function. This occurs, for instance, through degradation and turnover of both pre- and post-synaptic substrates, including synaptic vesicles, scaffold proteins, and neurotransmitter receptors [23,24,25]. In keeping with this, failure of mTOR-dependent autophagy was recently shown to promote aberrant synaptic clustering of GABA_A_ receptors and subsequent imbalance of excitation–inhibition in the brain, which might be key for epileptogenesis [26]. Alterations in mTOR-dependent autophagy are also implicated in abnormal dopamine system activity, which is implicated in epileptogenesis as well [27]. In this context, synaptic plasticity, besides being modulated by classic CNS molecules, is strongly affected by the immune system, and vice versa. This is due to a bidirectional communication and common molecular pathways that operate at the crossroad between the nervous and immune systems [28]. This is also the case for mTOR-related cell-clearing systems, which handle lymphocytes’ and other immune cells’ metabolism as well as antigen processing within both peripheral and CNS-resident antigen-presenting cells (APCs) [28]. Alterations in mTOR-related cell-clearing systems may lead to defective or inappropriate communication between the immune and nervous system, giving rise to a chain of inflammatory/immune and synaptic alterations, which may contribute to neurodevelopmental, neurological, and autoimmune diseases associated with seizures [28]. As support to these findings, increasing evidence indicates that beyond rapamycin, a variety of compounds that are known to induce autophagy may offer beneficial effects in epilepsy, including that associated with autoimmune disorders such as SLE [29,30,31,32,33].

Still in this context, it is worth mentioning that beyond autophagy, the ubiquitin–proteasome system (UPS), which regulates neuron excitability, synaptic plasticity, and neuro-inflammation/immunity, is altered in epilepsy as well [34,35,36,37]. It is remarkable that, similar to autophagy failure, UPS alterations in epilepsy are bound to mTOR hyperactivation [37]. This is in line with recent evidence documenting the role of mTOR in modulating both cell-clearing systems and the morphological convergence of autophagy and UPS [38,39,40]. In the present manuscript, we provide an update on the role of mTOR in epileptogenesis while discussing possible biochemical and molecular mechanisms bridging alterations of cell-clearing systems with epileptogenesis and epilepsy-induced neuronal alterations. The findings discussed here suggest that mTOR-dependent autophagy and UPS play a key role in epilepsy by producing plastic changes in the brain which may partially overlap with those responsible for neuroprotection against excitotoxicity.

## 2. mTOR and Autophagy in Human and Experimental Epilepsy

### 2.1. The Emerging Role of mTOR-Dependent Autophagy in the Regulation of the GABAergic System

Deficits in the number, migration, and positioning of cortical GABAergic inhibitory interneurons may lead to an imbalance of excitatory–inhibitory activity associated with overlapping neurological and neurodevelopmental disorders including epilepsy and autism [41,42,43].

Recent studies unraveled a role for mTOR/autophagy in regulating interneuron progenitors in the developing ventral telencephalon [44]. Conditional deletion of mTOR and subsequent autophagy activation reduces the number of interneurons in the cerebral cortex. This occurs in the absence of alterations of cortical interneurons’ positioning, suggesting a prominent role of mTOR-dependent autophagy in progenitor self-renewal rather than migration [44]. Intriguingly, in developing neural progenitors, a reciprocal regulation occurs between glycogen synthase kinase 3 (GSK3) and mTOR activity, which is likely aimed at finely tuning interneuron proliferation and migration [45]. The inhibition of GSK3 leads to elevation of mTOR activity, while overexpression of GSK3 suppresses mTOR activity. In turn, mTOR inhibition suppresses the hyperproliferation of GSK3-deficient neural progenitors. However, mTOR deletion per se does not produce any effects in radial migration of cortical projection neurons, at least during early-stage migration. This, in turn, might be due to a predominant role of AKT/PI3K/GSK3 pathway compared with mTOR during early cortical neuron migration [45]. It is remarkable that both mTOR and GSK3 are upstream negative regulators of autophagy, suggesting that autophagy is involved in both mTOR- and GSK3-dependent modulation of cortical neuron development and migration.

This is supported by evidence indicating that autophagy failure is bound to alterations of Rac1 protein [46], which is negatively regulated by mTOR, and it is implicated in epileptogenesis through regulation of interneuron migration. In detail, the deletion of Rac1 protein impairs the migration of postmitotic interneurons, leading to the early onset of spontaneous epileptic seizures [47,48]. Rac1 deletion causes generalized hyperactivity and cognitive impairment due to higher excitability and reduced spontaneous inhibitory currents in the Cornus Ammonis (CA) hippocampal pyramidal neurons [48]. Intriguingly, in TSC2-deficient cells featuring mTOR hyperactivation, Rac1 accumulates within cytoplasmic dots colocalizing with p62 and ubiquitin reminiscent of engulfed autophagy vacuoles, suggesting an impaired autophagy flux. Conversely, rapamycin administration activates Rac1 by dispersing its cytoplasmic aggregation [46]. Thus, autophagy activation rescues Rac1, which indirectly suggests that autophagy induction may target epileptogenesis associated with mTOR-dependent Rac1 dysfunction.

In a similar fashion, autophagy suppression due to TSC2-related mTOR hyperactivation disrupts the trafficking of GABA_A_ receptors via the sequestration of γ-aminobutyric acid receptor-associated proteins (GABARAPs) by p62-positive aggregates [26]. Such an effect is reproduced by autophagy deficiency following either ATG7 or ULK2 deletion in forebrain inhibitory or excitatory neurons [26,49]. In fact, beyond LC3, proteins belonging to the GABARAP family have been shown to be involved in the trafficking of GABA_A_ receptors to the neuronal plasma membrane, and they also have numerous binding partners that are involved in synapse formation, maintenance, and plasticity [23,50]. Following autophagy failure, elevated p62 sequesters a higher proportion of GABARAP thus limiting the amount of GABARAP available for GABA_A_ receptor surface presentation, eventually leading to imbalanced excitatory–inhibitory neurotransmission.

**Figure 1:**
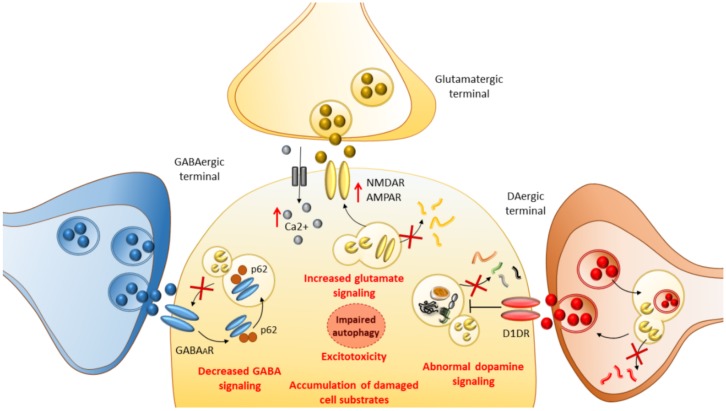
The role of autophagy in seizure-related molecular mechanisms. By operating at the level of gamma-aminobutyric acid (GABA), dopamine (DA) and glutamate systems autophagy are critically implicated in the molecular mechanisms underlying epileptogenesis and seizure-induced neuronal alterations. When a failure of autophagy occurs, elevated p62 hinders GABA_A_ receptor surface presentation, leading to decreased GABA signaling. At the same time, a failure of autophagy occludes the degradation of glutamate receptors N-Methyl-D-Aspartate (NMDAR) and A-Amino-3-Hydroxy-5-Methylisoxazole-4-Propionic Acid (AMPAR), fostering abnormal glutamate signaling and Ca^2+^ influx. This eventually leads to imbalanced excitatory–inhibitory neurotransmission underlying epileptogenesis. At the level of DA terminals, autophagy is seminal to blunt DA release by degrading DA-filled synaptic vesicles. Autophagy failure leads to abnormal DA release and abnormal stimulation of DA D1 receptors (D1DR), which in turn exacerbate autophagy suppression via mTOR hyperactivation. This eventually leads to the accumulation of damaged cell substrates which synergize with glutamate-related excitotoxicity to produce neuronal damage.

The present data indicate a novel physiological role for autophagy in regulating GABAergic signaling, providing a potential mechanism for the reduced inhibitory inputs observed in neurodevelopmental and neurological disorders such as epilepsy and autism [26]. These findings have bridged the gap between mTOR, autophagy, and GABAergic signaling, which started to emerge in 2009 when the role of autophagy in GABA_A_ receptor trafficking was first demonstrated [51]. In summary, autophagy-dependent synaptic clustering of GABA_A_ receptors during synaptogenesis is important for the function of inhibitory synapses, influencing synapse strength and, consequently, the balance of excitation and inhibition in the brain. Importantly, autophagy is implicated in neurotransmitter release as well as the internalization and endolysosomal turnover of neurotransmitter receptors beyond GABAergic ones (Figure 1). For instance, autophagy induction following inhibition of either GSK3β or mTOR regulates dopamine and glutamate release as well as the internalization of glutamate receptors [24,52,53,54,55]. The latter is associated with a decrease in the amount of intracellular Ca^2+^ and reduced excitotoxicity [55,56,57,58], which are key events implicated in seizure-induced neuronal damage (Figure 1).

### 2.2. Malformations of Cortical Development, TSC and FCD

Loss-of-function mutations in the two upstream mTOR regulators *TSC* and *PTEN* lead to hyperactivation of mTOR, which correlates with a high incidence of epilepsy in both humans and animal models [8,9,10,11,12,13,14,21,22,59,60,61,62,63]. The neurological syndromes associated with loss of *PTEN* or *TSC1/2*, including TSC and FCD, are collectively known as mTORopathies. TSC is an autosomal dominant disorder featuring severe neurological manifestations, including a high incidence (60%–90%) of early-onset, intractable epilepsy [60,61]. Typical pathological changes include cortical tubers, subependymal nodules, and subependymal giant cell astrocytoma (SEGA) [59,60,61,62,63]. Similar pathological changes are seen in the cortical malformations that characterize patients with FCD, a localized malformation of cortical development representing the commonest cause of severe childhood epilepsy [60,61]. Aberrant mTOR hyperactivation through autophagy impairment is widely implicated in epileptogenesis [12,13,21,22]. In fact, conditional deletion of *TSC1* and *PTEN* in mice forebrain neurons leads to severe seizures and increased mortality, which is associated with impaired autophagy activity [21]. These findings are recapitulated in human TSC and FCD brain specimens [21,22]. In fact, tubers and SEGA from TSC patients display a significant increase in phosphorylation of Ulk1 at Ser757 along with an increase of phosphorylated S6 and accumulation of the cargo autophagy protein p62. As shown by immunohistological staining, p62 accumulation occurs in morphologically distinct cell types, including enlarged, dysmorphic, giant ganglion-like cells in cortical tubers and spindle-shaped astroglial-like cells in SEGA. Similarly, balloon cells in FCD specimens contain prominent lysosomes due to a lack of fusion with autophagosomes along with an abnormal accumulation of p62. In both TSC and FCD, autophagy defects and epilepsy-related neuronal alterations can be, in part, reversed by in vitro inhibition of mTOR, suggesting that abnormal activation of mTOR may contribute to epileptogenesis through autophagy suppression in FCD and TSC [21,22].

Indeed, ablation of ATG7, specifically in relatively mature neurons within the cortex and hippocampus of mice, produces per se spontaneous recurrent seizures, which are even more severe than those induced by the ablation of *TSC1* and *PTEN* [21]. At the immunohistological level, autophagy suppression following ATG7-KO is reminiscent of what is observed in human TSC and FCD brains. In fact, there is a marked accumulation of p62 throughout the forebrain, with a pronounced increase in cortical neurons and hippocampal CA1 and CA3 pyramidal cells, and within the dentate gyrus (DG). Autophagy suppression correlates with a marked development of spontaneous seizures occurring mostly around postnatal weeks 8–10, along with an abnormal electrographic activity and reduced animal survival [21]. The present findings suggest that epileptogenesis is not restricted to a developmental time point; rather, it seems to be a continuous process throughout the life course. This hypothesis is also clinically relevant for those TSC patients who develop seizures during adolescence or adulthood [64]. Thus, targeting the mTOR/autophagy pathway could still be effective at a later point in life in TSC patients or in those with a later onset of seizure manifestation.

### 2.3. Autism Spectrum Disorders

The autophagy machinery is key for both synapse formation and pruning during development [65,66]. Autophagy suppression due to mTOR hyperactivation is implicated in aberrant synapse formation and refinement in autism spectrum disorders (ASD), which are associated with epilepsy [67]. In detail, mTOR hyperactivation in mice leads to spine pruning deficits in cortical projection neurons, indicating that autophagy is essential for synaptic pruning and that defects in autophagy contribute to autism-spectrum-like phenotypes [68].

Defects in autophagy machinery have been associated with aberrant axonal homeostasis underlying seizure-associated neurodevelopmental disorders. In detail, de novo pathogenic mutations in the autophagy gene *WDR45* (a mammalian homolog of yeast ATG18), which encodes a WD40 repeat-containing PtdIns(3)P binding protein, are detected in patients with developmental and epileptic encephalopathies [19,20]. For instance, *WDR45* mutations leading to impaired autophagy flux and accumulation of aberrant autophagy structures are detected in static encephalopathy of childhood with neurodegeneration in adulthood [19]. *WDR45* mutations in mice lead to cognitive impairment and defective axonal homeostasis, due to the accumulation of p62 and ubiquitin-positive protein aggregates which recapitulate some hallmarks of beta-propeller protein-associated neurodegeneration (BPAN) featuring adolescent-onset dementia, dystonia, and seizures [69].

Autism-like behavior with increased seizure propensity is reported in female patients with a heterozygous deficiency in the key autophagy gene Ambra1 [70]. Remarkably, Ambra1+/- mice which possess impaired autophagy show increased pentylenetetrazole (PTZ)-induced seizure propensity and a dysbalance of neuronal excitation-inhibition readout. The key role of autophagy in epileptogenesis underlying ASD is recapitulated in experimental fragile X syndrome (FXS) featuring a loss of neuronal fragile X mental retardation protein (FMRP) which is bound to mTOR overactivation and autophagy suppression [71]. FMRP deficiency leads to increased neuronal excitability and susceptibility to an epilepsy syndrome overlapping with benign focal epilepsy of childhood (benign rolandic epilepsy; BFEC) [72]. In mice models of FXS, it was recently demonstrated that autophagy is key in synaptic plasticity by degrading the synaptic scaffolding protein PSD-95, which is critical to spine morphology, and the immediate early gene Arc, which is critical for CA1 synapses plasticity [71]. In FMRP-KO neurons, mTOR hyperactivation and suppression of autophagy are bound to reduced degradation of PSD-95 and Arc, leading to an overabundance of filopodial-like spines, exaggerated long-term depression related to glutamate metabotropic receptors (mGluR-LTD), and impaired cognition. Acute knockdown of Raptor, a binding partner of mTORC1, activates autophagy, thereby enabling autophagy-dependent degradation of PSD-95 and Arc. This, in turn, corrects spine morphology, as well as mGluR-LTD at CA1 synapses and cognition in FMR1-KO mice. While it is well established that increased protein synthesis contributes to elevated PSD-95 and Arc, these findings document an additional role for impaired protein degradation in neurodevelopmental disorders [71]. These findings provide a potential mechanism by which down-regulated autophagy can lead to aberrant spine structure and synaptic plasticity at CA1 synapses, which may be relevant for seizure-associated disorders.

### 2.4. Astrocytomas

Epilepsy often develops in patients with high-grade astrocytoma (glioma), and the two conditions share common pathogenic mechanisms [73]. These include, for instance, altered expression of glutamate transporters and increased concentrations of extracellular glutamate, which contribute to epileptic discharge, tumor proliferation, and peripheral excitotoxicity. Again, hyperactivation of the mTOR pathway and concomitant autophagy suppression is a hallmark in the development and progression of high-grade astrocytoma [74]. In support of this overlap, antitumor therapy can contribute to seizure control, and antiepileptic drugs might have beneficial effects on tumors. This is the case of mTOR inhibitors and in particular Rapalogues. Discontinuation of Everolimus (prescribed for growing subependymal giant cell astrocytomas) is associated with seizure relapse, and seizure control is regained after reintroducing the drug [75]. Thus, clinical data support the potential of mTOR inhibitors in treating epilepsy in brain tumors.

### 2.5. Lafora Disease

Lafora Disease (LD) is the most frequent form of a group of epilepsies named progressive myoclonic epilepsies (PME). In fact, LD is an autosomal recessive disorder characterized by epileptic seizures, progressive myoclonus, and neurodegeneration, which is associated with the massive accumulation of polyglucosan inclusion bodies (Lafora bodies) [76]. LD is due to defects in either the laforin protein phosphatase or the malin ubiquitin ligase. The occurrence of inclusion bodies within different tissues indicates altered cell clearing pathways in LD. Accordingly, normal laforin is critical to promote autophagy progression [77], while the laforin–malin complex promotes degradation of intracellular misfolded proteins also by activating the UPS [78]. Thus, both laforin- and malin-KO cells display mTOR-dependent autophagy defects and reduced UPS activity [78]. The interconnection between LD and autophagy impairment has been proposed by several studies [79,80,81]. As shown in mice lacking laforin, autophagy impairment is considered as the primary trigger in LD [80,81]. This might be correlated with an early impairment of hippocampal and cortical GABAergic neurons, which anticipates the appearance of LD [82,83]. Recently, the autophagy-related transcription factor FoxO3a was identified as a possible cause for autophagy suppression in cellular and animal models of LD. In detail, the expression levels of FoxO3a and its targets LC3II and ATG12 are reduced in laforin-deficient cells and mice. Since FoxO3a exerts a negative control over mTOR, its loss could lead to autophagy defects associated with laforin deficiency [84].

### 2.6. Antiphospholipid Syndrome and Systemic Lupus Erythematosus

**Figure 2:**
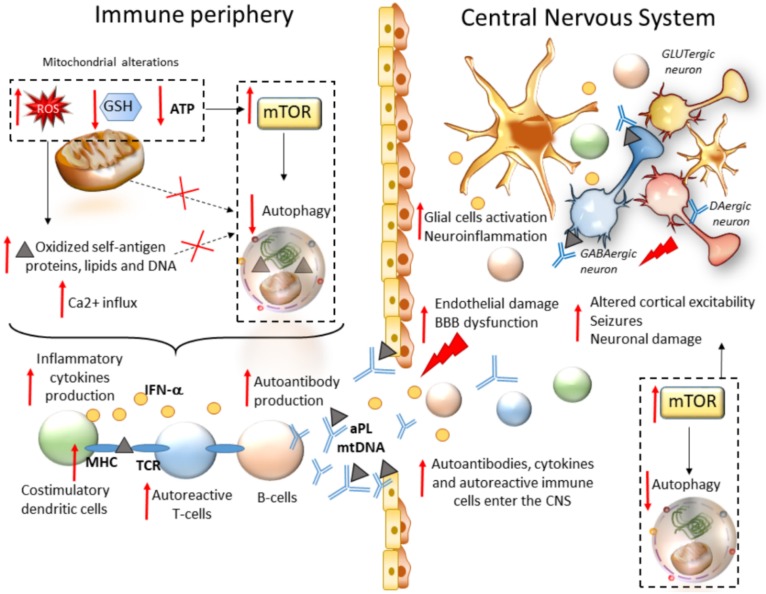
mTOR-dependent seizure development in autoimmune systemic disorders. Within peripheral immune cells, mitochondrial alterations lead to chronic oxidative stress (production of reactive oxygen species (ROS)) along with a concomitant depletion of antioxidant factors (gluthatione, GSH). This promotes oxidation of self-antigen proteins, lipids, and DNA, along with calcium (Ca^2+^) influx and the release of highly diffusible oxidative and inflammatory factors, which are spread to other intracellular organelles and through the bloodstream. At the same time, redox imbalance induces mTOR activation and autophagy impairment, eventually leading to impaired removal of oxidized self-antigens and mitochondria, production of inflammatory cytokines by costimulatory dendritic cells, increased stability of major histocompatibility molecules (MHC) on dendritic cells, subsequent activation of autoreactive T cells and production of autoantibodies by B cells. Autoantibodies, activated immune cells, and proinflammatory cytokines are spread through the bloodstream, and they reach the CNS where they produce endothelial damage and disruption of the blood–brain barrier (BBB). Within the brain milieu, these factors, coupled with mTOR hyperactivity and autophagy, impairment promote a chain of events consisting of neuroinflammation through activation of glial cells, altered cortical excitability, and eventually neuronal damage.

Chronic seizures often result from autoimmunity. This is best exemplified by antiphospholipid syndrome (APS) and SLE, prototypical autoimmune diseases that involve the CNS in most patients [85]. SLE is a prominent cause of seizures, which often represent an initial manifestation of the disease [86]. As recently addressed, mTOR pathway activation is a critical driver of inflammatory lineage development in autoimmune diseases including SLE [16]. In T cells from both SLE patients and animal models, oxidative stress along with a concomitant depletion of antioxidant factors occurs, which leads to a redox-dependent activation of mTOR. In turn, oxidative stress and mitochondrial dysfunction in T cells promote the release of highly diffusible inflammatory lipid hydroperoxides [87]. Oxidative stress is spread to other intracellular organelles and through the bloodstream, fostering modification of self-antigen proteins, lipids, and DNA, and eventually, development of autoimmunity. Thus, in autoimmune disorders such as SLE, oxidative stress may be key in bridging mTOR activation and immunogenicity of phospholipid antigens [88]. In SLE, abnormal T cell activation is associated with metabolic and organelle homeostasis, especially the mitochondrial, endosomal, and autophagy compartments [89]. This is not surprising since mTOR-dependent autophagy governs key metabolic cascades that dictate T- and B-cells’ differentiation, function, and activity [90,91]. Autophagy is also key in adaptive immunity, being implicated in the major histocompatibility class II (MHC-II)-restricted presentation of exogenously derived antigens to CD4+ T cells [92,93]. Autophagy also handles MHC-I internalization and degradation, thus influencing MHC-I stability at the plasma membrane of antigen-presenting cells (APCs), and subsequent CD8+ T-cell responses [94,95]. In fact, autophagy inhibition within APCs occludes the surface internalization of MHC-I molecules, leading to autoimmunity due to increased endogenous antigen presentation and stimulation of autoreactive T-cells [96]. This is intriguing since mTOR activation, and likely autophagy alterations, underlie the generation of antiphospholipid antibodies (aPL), which are mediators of seizure in adults and children alike [17,18]. Remarkably, aPLs from APS patients are able to gain access to the CNS following injection in mice, suggesting that they may play a direct role in the pathogenesis of neurological manifestations in APS/SLE [97]. Despite not being fully characterized, the mechanisms leading to neurological manifestations may include aPL-induced micro- and macro-thrombosis, alterations of brain–blood barrier and immune-mediated neuronal toxicity in the brain [98]. As far as it concerns the pathogenesis of epilepsy, it has been shown that exogenously administered IgG–aPL from APS patients induces depolarization of mouse brain synaptoneurosomes [99]. aPLs were recently shown to reduce autophagolysosomal proteolysis in monocytes, suggesting that dysregulation of autophagy may be bound to monocytes hyperactivation in APS/SLE [100]. Similarly, mTOR-dependent autophagy impairment was documented within SLE monocytes following sustained inflammatory stimuli such as IFNα. This, in turn, is associated with enhanced mitochondrial damage, oxidative stress and accumulation of undigested mitochondrial DNA (mtDNA) which is released extracellularly to trigger anti-DNA autoimmunity [101]. Accordingly, polymorphisms in the ATG5 gene are associated with disease susceptibility in SLE patients, with the rs2245214 polymorphism being significantly associated with a higher risk of producing anti-DNA autoantibodies [102]. In mice models, defects in LC3-associated phagocytosis, a noncanonical form of autophagy, cause SLE-like phenomena including increased serum levels of inflammatory cytokines and autoantibodies [103]. Contrariwise, the mTOR blockade has remarkable therapeutic benefit in mice and patients with SLE [32,33,104]. In SLE patients, rapamycin treatment normalizes cytosolic and mitochondrial Ca^2+^ levels and T-cell-activation-induced Ca2+ fluxing, which are known to contribute to the inflammatory process in SLE [33]. As thoroughly reviewed elsewhere, rapamycin also limits the proliferation and activity of autoreactive T cells by promoting regulatory T cell and tolerogenic dendritic cell expansion, and it blunts proinflammatory IFN-α production by plasmacytoid dendritic cells and blunts T cell stimulation of autoreactive B cells in SLE [7]. Thus, mTOR inhibitors, and potentially autophagy activators, may influence seizure development by acting outside the CNS through modulation of proinflammatory linage development (Figure 2) [88]. mTOR-dependent autophagy alterations may be also implicated in the generation of self-antigens which reach the CNS to produce alterations of cortical excitability, though this remains to be specifically demonstrated (Figure 2).

### 2.7. Experimental Models of Acquired Epilepsy

Common brain injuries, such as status epilepticus (SE), stroke/ischemia, and neurotrauma, are associated with acquired epilepsy, which develops in three phases: (i) the injury (CNS insult), (ii) epileptogenesis (latency), and (iii) the chronic epileptic (spontaneous recurrent seizure) phases [2]. Most experimental models are set up to mimic temporal lobe epilepsy (TLE), the most common form of epilepsy comprising two-thirds of patients with intractable seizures. These models produce seizures that originate from the hippocampus and/or limbic cortical areas, and they include lesions such as excitotoxic SE, electrically induced seizure, and traumatic brain injury, as well as induction of inflammatory processes by hyperthermia and viral inflammation, among others [11]. Two well-known models consist of the systemic administration of chemoconvulsants like kainic acid (KA, an agonist of glutamatergic non-NMDA receptors), or pilocarpine (PILO, an acetylcholine muscarinic receptor agonist). In rodents, these models produce limbic seizures, which are secondarily generalized and can be easily scored in terms of behavior (through specific behavioral scales), electroencephalographic (EEG) features, and duration [12]. In these models, a neuropathological alteration reminiscent of human TLE occurs, namely mesial temporal sclerosis or Ammon’s Horn Sclerosis [12,105]. It consists of the loss of pyramidal cells of the CA areas CA1 and CA3–CA4, along with the loss of interneurons of the hilus of the DG, and aberrant proliferation of mossy fibers originating from DG granule cells, which is known as mossy fiber sprouting [106].

Other epilepsy models exist as well which bear interesting features for studying epileptogenesis. For instance, pentylenetetrazole (PTZ) is a GABA_a_ receptor antagonist used to induce seizures in experimental settings through decreasing inhibitory GABA activity. PTZ’s effect is most prominent in the cortex and hippocampus, and its acute administration is a valuable model of generalized tonic-clonic and myoclonic seizures [107,108], while repetitive administrations of PTZ (kindling) are needed to induce SE and pathological alterations [109,110]. Chemical kindling seizures triggered by PTZ are assumed to mimic the pathogenesis of human epilepsy and are considered a model of drug-resistant epilepsy [109,110]. In the following sections, we discuss the role of mTOR/autophagy in various experimental models of acquired epilepsy.

#### 2.7.1. Chemoconvulsive (Kainite and Pilocarpine) and Electroconvulsive Status Epilepticus

KA-induced seizures produce a biphasic activation of mTOR [111]. In detail, an increase in P-S6 expression occurs at 1 h after seizure onset, reaching a peak at 3-6 h and returning to baseline by 24 h in the hippocampus and neocortex of mice. After resolution of SE, a second increase in P-S6 occurs in the hippocampus starting at 3 days, peaking at 5–10 days, and persisting for several weeks after KA injection. Remarkably, such a persistent increase correlates with the development of chronic epileptogenesis [111]. Pretreatment with rapamycin through inhibition of mTOR abolishes both the acute and chronic phases of KA-induced seizures, neuronal cell death, and mossy fiber sprouting. When rapamycin is administrated after the termination of SE, it reverses only the chronic phase of mTOR activation while reducing mossy fiber sprouting and epilepsy without any evident effects on neuronal death [111]. Similar results on mossy fiber sprouting were obtained in a model of PILO-induced status epilepticus [112]. Focal infusion of rapamycin for 1 or 2 months into the dorsal hippocampus after SE reduces mossy fiber sprouting. These effects progressively disappear following rapamycin withdrawal. Thus, mTOR inhibition, at least in PILO-induced SE, may not be sufficient per se to persistently prevent the onset of epilepsy; rather, long-term rapamycin exposure may be mandatory to prevent seizure onset. These findings are recapitulated under the term *epileptostatic* for describing the mechanisms through which rapamycin may interfere with the effects of epileptogenic insults [11,14].

In murine models of KA-induced seizures, the deletion of mTOR from 44% of the astrocyte population leads to a lower seizure frequency while ameliorating astrogliosis in the sclerotic hippocampus compared with controls [113]. These effects are bound to mTOR-dependent increased stability of the astroglial glutamate transporter 1 (Glt1) and subsequent enhanced extracellular glutamate removal by astrocytes.

A correlation between mTOR-dependent protection against blood–brain barrier disruption and seizure severity was observed in a rat model of electrically induced SE through angular bundle stimulation [114]. In detail, rats chronically administered with rapamycin starting 4 hours after electrically induced SE are refractory to develop spontaneous seizure, and they are partially protected from neuronal loss and mossy fiber sprouting. These beneficial effects correlate with reduced blood–brain barrier leakage compared with controls, though the mechanism by which rapamycin maintains the blood–brain barrier integrity remains to be elucidated [114].

Contrary to the consensual results on mTOR hyperactivation, the role of autophagy in SE appears puzzling, yet as a double-edged sword. In hippocampal extracts from KA-treated mice, the levels of phospho-mTOR increase from 6 to 16 h, while phospho-Akt increases at 16 h following KA treatment [115]. Intriguingly, a significant increase in the amount of LC3-II is detected at 4–6 h following KA, though such an effect is not paralleled by alterations in ATG5, ATG6, and ATG7 levels. These data were interpreted as a transient induction of autophagy attempting to face excitotoxic cell death in the mouse hippocampus [115]. In line with this, KA-induced seizures also cause an immediate, though transient, vacuolization of astrocytes, which precedes astrogliosis [116]. These events are prevented by pre- or post-treatment with rapamycin, indicating a transient induction followed by a progressive impairment of autophagy flux which cannot cope with frank astrogliosis.

An upregulation of LC3-II has been similarly detected in the hippocampus of rats with lithium-PILO-induced SE [117]. In order to exclude that the increase in LC3-II expression was due to stagnant autophagosome accumulation or to a reduction in lysosomal activity, parallel evaluation of the expression of LC3 and the lysosomal marker LAMP1 was monitored. At 24 h post-SE, LAMP1 was highly expressed, and accumulated along with LC3-positive-dots within DG mossy fibers [117]. In line with these findings, in a mice model of SE with electroconvulsive seizures occurring in the absence of neuronal loss, increased levels of ATG5–ATG12 and LC3-II were detected in the hippocampus, the dentate gyrus, and the CA [118]. Whether such an autophagy increase might contribute to exacerbating SE, or whether it might occur as a compensatory attempt to survive the epileptic insult by coping with synaptic and neuronal rearrangements, is still a matter of debate and ongoing investigation.

In support of the second hypothesis, chronic overactivation of glutamate receptors leads to autophagy impairment due to the blockage of autophagy progression [119]. Indeed, despite producing an increase in autophagy markers, excitotoxic glutamate produces a late-stage block of autophagy in vivo in hippocampal neurons [120]. Consistently, excitotoxic neuronal cell death is worsened by autophagy blockers while it is prevented by autophagy inducers [121,122,123].

Again, it has been documented that phospholipase D (PLD), which acts as an inhibitor of mTOR/AMPK-autophagy [124,125], is implicated in KA-induced seizures [126]. In detail, PLD is a downstream target of the GTPase Rheb, which is inhibited in response to AMPK via TSC [124]. In fact, the inhibitory effects of PLD on AMPK activity are mediated by mTOR, and a reciprocal feedback mechanism involving AMPK and mTOR is involved in PLD-mediated autophagy suppression [124]. PLD suppresses autophagy by modulating mTOR and AMPK-dependent phosphorylation of ULK1 and also by suppressing the interaction of Beclin-1 with vacuolar-sorting protein 34 (Vps34) [125]. Conversely, PLD inhibition enhances autophagy flux, indicating that PLD coordinates major players of the autophagy pathway [125]. Remarkably, the levels of both PLD1 and PLD2 isoenzymes are increased in the rat hippocampus after KA-induced seizures [126]. PLD1 immunoreactivity following KA injection is preferentially increased within the CA3 and CA1 subregions, and this occurs mostly within reactive astrocytes, likely in response to brain insults at the late stage when remodeling occurs. Likewise, PLD2 expression in reactive astrocytes persists at 10 d after KA injection. On the other hand, PLD2 increase within neurons peaks at 1 to 3 d and returns at baseline 10 d after seizure insult. Intriguingly, aberrant neuronal expression of PLD2 is restricted mainly to the infrapyramidal blade of the DG. This is in line with studies demonstrating that after KA-induced seizures, the sprouted axon collaterals from granule cells in the infrapyramidal blade cross the hilus and project into the supragranular layer of suprapyramidal blade, but not vice versa [127]. Thus, PLD2 expression in the infrapyramidal blade of the DG may play a role in the characterization of the spatial distribution of the sprouted mossy fiber pathways after epileptic seizures [126]. Since PLD overexpression is bound to autophagy suppression via mTOR upregulation, it would be worth investigating whether a causal relationship exists between PLD and autophagy alterations in seizure-induced alterations within the infrapyramidal blade of the DG.

In murine models of PILO-induced SE, mTOR-dependent autophagy was recently associated with the beneficial effects of several phytochemical compounds such as curcumin (*C. Longa*), *Ginkgo biloba* L. (Ginkgoaceae), and Aucubin (*Eucommia ulmoides* Oliv.). Treatment with *Ginkgo biloba* reduces seizure severity score, and it improves spatial cognitive functions and recognition memory while protecting against neuronal damage and mossy fiber sprouting in the DG and CA. These effects occur along with a reduction of hippocampal mTOR and its downstream ribosomal S6 and pS6 protein levels [128]. Likewise, in models of PILO-induced status epilepticus, either aucubin or curcumin protects from post-SE cell death within DG, Hilus, CA1, and CA3 hippocampal regions through autophagy activation and inhibition of necroptosis [29,30].

In recent years, lithium, a classic mood stabilizer and autophagy inducer, was shown to produce neuroprotective effects in many neurological diseases, including epilepsy. The effects of systematically administered lithium on PILO-induced seizure activity, susceptibility, and severity are dose-dependent [31]. In detail, while high-dose lithium (40 mg/kg) increases the susceptibility and severity of PILO-induced seizures, low-dose lithium (10 mg/kg) administered to PILO-treated rats markedly decreases the proportion of Racine stage 4–5 seizures, it extends latency until seizure onset and significantly reduces the frequency of lower-class seizures [31]. Despite the autophagy-related effects of lithium not being examined, these findings provide a framework for further investigating the implication of autophagy in the electrophysiological mechanisms of excitatory and inhibitory imbalances within neural circuits which regulate seizure activity.

#### 2.7.2. Pentylenetetrazole (PTZ)-Induced Seizures

Contrary to models of chronic epilepsy such KA, which exhibit biphasic mTOR pathway activation, PTZ seizures were shown to produce only acute mTOR activation, while administration of PI3K inhibitors prevents PTZ acute epileptogenesis [108]. In PTZ-treated larval, juvenile, and adult zebrafish, rapamycin pretreatment slows down the progression of seizures by prolonging the latency to reach the tonic–clonic stage (stage III) [129]. Similar results are reported in rat models of PTZ-induced kindling, which feature mTOR hyperactivation [130]. In detail, a massive increase in phospho-mTOR and its downstream targets occurs in the hippocampi of PTZ-treated rats. This is attenuated by pretreatment with adenosine, acting as an alternative, endogenous anticonvulsant [130]. Adenosine-induced suppression of mTOR and S6 phosphorylation can be reversed by the application of compound C, an inhibitor of AMP-activated protein kinase (AMPK), which acts as an upstream suppressor of the mTOR pathway and autophagy activator. This suggests that autophagy impairment due to mTOR hyperactivation and AMPK downregulation may be implicated in PTZ-induced kindling [130].

The role of autophagy in PTZ-induced SE was recently confirmed by a study evaluating the potential therapeutic effects of endothelial progenitor cells (EPCs) in sustained seizures [110]. EPCs may confer therapeutic effects against epilepsy and its associated behavioral and biochemical abnormalities, at least in part, via the upregulation of autophagy. In detail, intravenously administered EPCs home into the hippocampus, where they mitigate PTZ-induced behavioral abnormalities, neurological damage, and histopathological alterations, as well as perturbations in neurotransmitter activity. These effects correlate with an increase in LC3, Beclin-1, and ATG7 levels, and they are reproduced by the administration of the antiepileptic drug valproic acid [110], which is known to induce autophagy through mTOR/Akt inhibition [131].

Autophagy activation is also detected following ibuprofen administration in PTZ-treated rats, which is associated with reduced seizure number, duration, and severity, along with decreased astrogliosis and astrocyte proliferation [132]. Likewise, autophagy is implicated in the beneficial effects of the ketogenic diet (KD) in PTZ-kindled rats [133]. KD alleviates seizure severity while decreasing the number of apoptotic cells and the number of damaged mitochondria in the hippocampi of kindled rats. These effects correlate with autophagy activation as evidenced by an increase of hippocampal Beclin-1, ATG5, and LC3-II/ LC3-I ratio along with a decrease of the autophagy substrate p62 compared with PTZ-kindled rats which are fed with normal diet [133]. Remarkably, these effects are abolished by pretreatment with the autophagy inhibitor 3-MA, while rapamycin pretreatment protects from neuronal cell death in the hippocampus despite not affecting seizure severity.

In a mice model of PTZ-induced seizures, protective autophagy-related effects were also reported for sitagliptin, a selective inhibitor of dipeptidyl peptidase (DPP)-4 that is commonly used in the treatment of type 2 diabetes [134]. Sitagliptin improves neurochemical alterations and protects against PTZ-induced neurotoxicity by i) enhancing hippocampal GABA activity and reducing glutamate activity; ii) producing anti-peroxynitrite, antioxidant, and anti-inflammatory effects; and iii) reducing caspase-3-related apoptosis while enhancing autophagy. In this context, the increase in autophagy activity is paralleled by Nrf2 activation, which is implicated in mitophagy and mitochondriogenesis beyond antioxidant defense [134]. The antiepileptic, protective, and autophagy-inducing effects of sitagliptin are enhanced upon coadministration with pregabalin, suggesting that autophagy holds center stage in the biochemical mechanisms underlying the antiepileptogenic potential of these compounds.

Similar to what is reported for PILO, the autophagy inducer lithium produces anticonvulsive and neuroprotective effects in murine models of PTZ-induced seizures [135]. While acute lithium administration (30 mg/kg) enhances the proconvulsive properties of PTZ, chronic pretreatment with lithium (7 days, 10 mg/kg) significantly increases the seizure threshold. At the same time, lithium confers neuroprotection in primary cerebellar cultured neurons by counteracting glutamate-induced excitotoxicity. In fact, both anti-epileptogenic and neuroprotective effects of lithium are reversed by the coadministration of an NMDA receptor antagonist [135]. It is remarkable that similar to the protective effects of mTOR inhibitors against the neurotoxic convulsant quinolinic acid [58], lithium protects against glutamate-induced neurotoxicity, and such an effect is associated with autophagy activation, mostly related to GSK3β inhibition [119,120,121,122,123]. Besides promoting neuronal survival following glutamate-induced injury, inhibition of either mTORC1 or GSK3β similarly prevents NMDA-induced decrease in spontaneous excitatory postsynaptic currents [55]. This suggests that the plastic effects of excitatory–inhibitory transmission dysbalance involve a common mechanism which requires the permissive activity of mTORC1 and GSK3β, likely converging in a failure of the autophagy pathway. This is in line with the abovementioned evidence indicating that a crosstalk occurs between GSK3β and mTOR during brain development [45]. TSC2 is key in mediating such a cross-talk since GSK3 directly binds to and phosphorylates TSC2 in neural progenitors [45]. This phenomenon, which may disclose novel autophagy-related regulatory pathways in epilepsy, remains poorly characterized and deserves further investigation.

#### 2.7.3. Ischemic/Hemorragic Stroke and Brain Trauma

Pre-ischemic hyperglycemia increases the occurrence of post-ischemic seizures both in clinical and experimental settings [136]. The development of post-ischemic seizures in hyperglycemic animals is associated with activation of mTOR and ERK1/2 pathways. Rapamycin treatment inhibits the post-ischemic generalized tonic–clonic seizures while protecting from neuronal death in hyperglycemic ischemic rats through inhibition of mTOR and ERK pathways [136].

Stroke is also implicated in the etiology of seizures development. In rat models of global cerebral ischemia (GCI), during the first and the second days following GCI, convulsive seizures frequently occur, which are accompanied by seizure discharge shown by the EEG [137]. Convulsive seizures occur along with an increased expression of phospho-mTOR and GLUT-1 in the cerebral cortex and hippocampus, as evidenced by immunohistochemistry and western blot analyses. Mild hypothermia and/or rapamycin treatment alleviates GCI-induced seizures by reducing the number of epileptic attacks, seizure severity scores, and seizure discharges [137]. This goes along with a reduction of phosphor-mTOR and its downstream effector p70S6 in neurons. Thus, mTOR is involved in stroke-induced seizures and targeting mTOR hyperactivation through rapamycin, and mild hypothermia produces antiseizure effects [137].

In a rat model of FeCl_2_-induced post-traumatic epilepsy (PTE), p-mTOR and p-P70S6K are increased significantly in the hippocampus and perilesional cortex [138]. Rapamycin administration decreases mTOR activity markers while reducing the frequency and number of behavioral seizures and epileptic brain injury [138].

The role of autophagy in cerebral ischemia, stroke, and brain trauma is still ambiguous, with numerous studies describing autophagy either as mediating neuronal death or protection [139,140,141]. To date, there is a paucity of studies investigating the role of autophagy specifically in epileptogenesis associated with these disorders. An exception is cerebral cavernous malformation (CCM), a major cerebrovascular disease characterized by enlarged and leaky capillaries that predispose one to seizures, focal neurological deficits, and fatal intracerebral hemorrhages [142]. Causative loss-of-function mutations associated with both sporadic and familial forms have been identified in three genes, namely *KRIT1* (CCM1), *CCM2* (MGC4607), and *PDCD10* (CCM3). Recently, it was demonstrated that the ablation of the *KRIT1* gene strongly suppresses autophagy, leading to the aberrant accumulation of the autophagy adaptor p62, defective quality control systems, and increased intracellular stress [142]. KRIT1 loss-of-function suppresses autophagy through activation of the mTOR-ULK1 pathway, while treatment with mTOR inhibitors rescues some of the molecular and cellular phenotypes associated with CCM [142]. Insufficient autophagy is also evident in CCM2-silenced human endothelial cells, in cells and tissues from endothelial-specific CCM3-KO mice, as well as in human CCM lesions [142]. Furthermore, defective autophagy is highly correlated to endothelial-to-mesenchymal transition, a crucial event that contributes to CCM progression.

#### 2.7.4. DA-Dependent Kindled Seizures

Repeated systemic administration of a type 1 DA receptor (D1DR) agonist (SKF81297) was shown to induce kindled seizures in mice [27]. Remarkably, this occurs along with hyperactivation of the mTOR signaling in the hippocampus. These effects are associated with disrupted long-term potentiation (LTP) in the DG and altered recognition memories. Conversely, rapamycin administration delays the development of SKF81297-induced kindled seizures, and it rescues LTP in the DG and object recognition [27]. This suggests that abnormal stimulation of DA D1 receptors (D1DRs) is sufficient to induce generalized seizures, leading to the overactivation of mTOR signaling, disrupted hippocampal plasticity, and impaired long-term recognition memories. Likewise, in experimental hepatic encephalopathy, abnormal stimulation of D1DRs alters synaptogenesis by producing a reduction of GABA_A_-induced currents due to the loss of interaction of GABA_A_ receptors with AKT, which is placed upstream of mTOR-dependent autophagy [143]. It is remarkable that mTOR-dependent autophagy is key to blunt abnormal electrically evoked DA release through the degradation of DA-containing synaptic vesicles [52]. Thus, autophagy is key to prevent both abnormal stimulation of D1DRs and dysfunction of GABA_A_ receptors, which indirectly suggests that autophagy induction may be key to regulate cortical excitability through modulation of DA–GABA interactions. These findings are also key in the context of epileptic seizures which sporadically occur following administration/intake of strong DA-releasing abused drugs/psychostimulants such as amphetamines. In fact, amphetamines produce an abnormal DA release featuring peaks and drops of extracellular concentration, which in turn lead to abnormal pulsatile stimulation of D1DRs in the brain [28,144]. It is remarkable that abnormal stimulation of D1DRs, as it occurs following amphetamine administration, leads to noncanonical intracellular pathways, which produce mTOR hyperactivation and autophagy suppression [145,146]. These, in turn, may produce maladaptive plastic changes including altered cortical excitability and abnormal NMDA and AMPA receptor stimulation up to glutamate-induced excitotoxicity [144].

## 3. A Glance at mTOR-Related UPS Alterations in Epilepsy

Recently, increasing evidence indicates a functional cross-talk between UPS and autophagy, which occurs at both biochemical and morphological levels [28,38,39,40,41,42,43,44,45,46,47,48,49]. This is largely due to the mTOR pathway, which modulates both UPS- and autophagy-dependent protein degradation [30]. This is not surprising since autophagy and UPS share most of their substrates and functions, and they operate coordinately in neurons and glia to modulate oxidative/inflammatory stress response, neurotransmission, and synaptic plasticity [28]. In fact, the inhibition of either UPS or autophagy in experimental models produces marked alterations in neurotransmitter activity, synaptic plasticity, and neurodegeneration [52,147,148,149]. mTOR-related UPS dysfunctions beyond autophagy alterations are now emerging in the field of epilepsy as well.

The coordinated turnover of synaptic membrane proteins via the UPS/autophagy–endolysosomal pathway is essential for synaptic function. For instance, hypomorphic mutations in the ubiquitin adaptor protein PLAA cause an infantile-lethal neurological syndrome with seizures in both humans and mice [150]. Due to impaired degradation of ubiquitinated substrates, PlAA mutant neurons accumulate K63-polyubiquitylated proteins and synaptic membrane proteins, disrupting synaptic vesicle recycling, neurotransmission, and excitatory–inhibitory balance.

A downregulation of ubiquitin carboxyl-terminal hydrolase isozyme L1 (UCHL1) was recently detected in murine models of SE induced by intra-amygdala KA injection [151]. UCHL1 inhibition prior to SE decreases hippocampal ubiquitin, and it disrupts UPS function and the turnover of PSD-45; moreover, it prolongs SE-induced seizures, and it attenuates the EEG response to anticonvulsant lorazepam while exacerbating seizure-induced cell death. Remarkably, rapamycin administration can reverse these effects by increasing UCHL1 expression in vivo. Thus, post-transcriptional loss of UCHL1 following SE is deleterious to neuronal survival and may contribute to epileptogenesis and epilepsy-induced brain damage, while mTOR inhibition can restore these alterations by rescuing UPS beyond autophagy [152]. Inhibition of the UPS also exacerbates epileptogenesis in experimental mesial TLE, causing early and frequent spontaneous seizures, neuron loss and aberrant mossy fiber sprouting [152]. The UPS participates in the pathogenesis of mesial TLE through a mechanism that involves the upregulation of Nedd4-2, a critical E3 ligase linked with ion channels and synaptic vesicle recycling. In fact, inhibition of ubiquitin enhances the activation of Nedd4-2, which in turn switches the α subunit epithelial sodium channel (α-ENaC) downstream, thus contributing to epileptogenesis [152].

The core machinery of the UPS, the 26S proteasome, is composed of β catalytic subunits existing as either constitutive or inducible isoforms. During inflammation or oxidative stress, the constitutive subunits of the P26S are replaced by their inducible counterparts, leading to the formation of the immunoproteasome, which is endowed with pathophysiological functions related to immunity and inflammation [35]. mTOR hyperactivation has been linked with downregulation of constitutive proteasome subunits and subsequent induction of immunoproteasome. In the healthy human brain, the expression of the catalytic inducible β5 (β5i) subunit of the immunoproteasome is almost absent, while it is markedly enhanced in epileptogenic foci from patients with pharmaco-resistant seizures [36]. The induction of β5i immuno-subunit in neurons and glia occurs even in experimental epilepsy, and its selective pharmacological inhibition significantly prevents, or delays, 4-aminopyridine-induced seizure-like events in acute rat hippocampal/entorhinal cortex slices [36]. Likewise, increased expression of (immuno)proteasome subunits (β1i, β5i) is detected in the post-SE rat model of TLE, in both neurons and astrocytes within the hippocampus and piriform cortex. Rapamycin-treatment in post-SE rats reduces (immuno)proteasome expression and the number of spontaneous seizures compared to vehicle-treated rats. (Immuno)proteasome expression is also increased in neurons and astrocytes within the human hippocampus after SE and in patients with drug-resistant TLE as well as TSC and FCD [37,122]. In vitro studies using cultured human astrocytes showed that interleukin (IL)-1β-induced (immuno)proteasome gene expression could be attenuated by rapamycin, supporting a role for altered mTOR-dependent cell-clearing systems as a potential target in epileptogenesis [37,153].

## 4. Conclusions

In the last decades, alterations of autophagy and UPS started to apply to the field of epileptology besides neurodegenerative disorders. In the present review, increasing evidence was presented indicating a link among both genetic and acquired forms of epilepsy with alterations in mTOR-dependent cell-clearing systems. Despite wide evidence suggesting a failure of autophagy in epileptogenesis and epilepsy-induced neuronal alterations, some controversies still exist which tone down viewpoints linking autophagy failure with most of the epilepsy-related mTOR alterations.

In keeping with this, the effects of rapamycin, which is employed as a gold-standard mTOR inhibitor and autophagy inducer, vary according to the dosing, timing, and experimental contexts [154]. Different doses of rapamycin are needed to suppress the phosphorylation of different mTOR substrates, and differential sensitivity of the two mTOR complexes mTORC1 and mTORC2 to rapamycin occurs [154]. The intriguing properties of rapamycin dosage are also bound to rapamycin’s competition with phosphatidic acid (PA) for mTOR. Since PA is a central metabolite of membrane lipid biosynthesis and the product of the PLD, which is increased in epilepsy, confounding outcomes cannot be ruled out. Besides these considerations, potential side effects of mTOR inhibitors need to be taken into account. Minimizing drug dosage to reduce side effects while maintaining therapeutic efficacy represents indeed a major goal. Thus, mTOR inhibitor treatment is aimed at restoring mTOR to control levels rather than completely shutting down its activity [155]. Extrapolating the absolute dosing and serum levels from mice to humans is challenging, though in TSC mice models, moderate doses of rapamycin (3 mg/kg/d) were shown to lower P-S6 at levels comparable with controls, while high doses (10 mg/kg/d) completely inhibit P-S6 expression [155]. Another approach to reducing drug exposure and side effects is intermittent dosing or drug holidays. Rapamycin treatment (3 mg/kg) with drug holidays of 24 days was shown to almost completely prevent epilepsy in TSC mice models [155]. Remarkably, the antiepileptogenic effects of intermittent rapamycin dosing outlast the duration of mTOR inhibition, indicating that the pharmacodynamic actions of mTOR inhibition persist beyond pharmacokinetic properties [155]. Another issue that deserves further attention is that the vast majority of studies employ LC3 quantification as a gold-standard assay for monitoring the autophagy status, which may have considerable methodological limitations. This is magnified with immunofluorescent microscopy, which can yield false-positive results due to the misdetection of LC3 puncta corresponding to cytosolic rather than authentic autophagy-related structures [156]. In fact, endogenous LC3-positive puncta are evident and become even larger in cells where autophagy is inhibited, questioning the reliability of LC3-immunofluorescence in cells and tissues with compromised autophagy [156]. In line with this, immune-gold-based electron microscopy remains seminal for detecting the ultrastructural LC3 compartmentalization within autophagy vacuoles, which is disrupted, for instance, by amphetamine administration [40]. In fact, despite being LC3-increased following amphetamine intoxication, it loses its polarization within autophagy-related structures [40]. Combining different techniques in order to reliably investigate the autophagy status needs to be applied to the field of epilepsy as well. Another issue which deserves to be investigated is the concomitant assessment of the contribution of each cell-clearing pathway considering the novel scenario where autophagy and UPS represent a unified mTOR-dependent cell-clearing apparatus. Overall, the findings discussed here encourage further studies aimed at investigating the therapeutic potential of mTOR inhibitors and autophagy inducers in seizure-related disorders.

## Abbreviations

FCD	Focal Cortical Dysplasia
FMRP	Fragile X Mental Retardation Protein
BFEC	Benign Focal Epilepsy of Childhood
LTD	Long-Term Depression
PME	Progressive Myoclonic Epilepsy
FXS	Fragile X Syndrome
GABARAP	γ-Aminobutyric-Acid-Receptor-Associated Proteins
GSK3	Glycogen Synthase Kinase 3
GABA	Gamma-Aminobutyric Acid
KA	Kainic Acid
PILO	Pilocarpine
NMDA	N-Methyl-D-Aspartate
AMPK	Amp-Activated Protein Kinase
EPCs	Endothelial Progenitor Cells
KD	Ketogenic Diet
DPP	Dipeptidyl Peptidase
GCI	Global Cerebral Ischemia
EGG	Electroencephalography
FeCl_2_	Ferrous Chloride
PTE	Post-Traumatic Epilepsy
CCM	Cerebral Cavernous Malformation
D1DR	type 1 dopamine Receptor
LTP	Long-Term Potentiation
AMPA	A-Amino-3-Hydroxy-5-Methylisoxazole-4-Propionic Acid
UCHL1	Ubiquitin Carboxyl-Terminal Hydrolase Isozyme L1
α-ENaC	α Subunit Epithelial Sodium Channel
DA	Dopamine
LD	Lafora Disease
mTOR	Mammalian Target Of Rapamycin
PTEN	Phosphatase And Tensin Homolog
PTZ	Pentylenetetrazole
SEGA	Subependymal Giant Cell Astrocytoma
CA	Cornus Ammonis
DG	Dentate Gyrus
ASD	Autism Spectrum Disorders
BPAN	Beta-Propeller Protein-Associated Neurodegeneration
TLE	Temporal Lobe Epilepsy
TSC	Tuberous Sclerosis Complex
UPS	Ubiquitin-Proteasome System
SLE	Systemic Lupus Erythematosus
APS	Antiphospholipid Syndrome
MHC-II	Major Histocompatibility Class-II
APC	Antigen Presenting Cell
mtDNA	Mitochondrial DNA
IFN-α	Interferon alpha
PLD	Phospholipase D
PA	Phosphatidic Acid
CNS	Central Nervous System
